# Toward a Machine Learning Predictive-Oriented Approach to Complement Explanatory Modeling. An Application for Evaluating Psychopathological Traits Based on Affective Neurosciences and Phenomenology

**DOI:** 10.3389/fpsyg.2020.00446

**Published:** 2020-03-24

**Authors:** Pasquale Dolce, Davide Marocco, Mauro Nelson Maldonato, Raffaele Sperandeo

**Affiliations:** ^1^Department of Public Health, University of Naples Federico II, Naples, Italy; ^2^Department of Humanistic Studies, University of Naples Federico II, Naples, Italy; ^3^Department of Neuroscience and Reproductive and Odontostomatological Sciences, University of Naples Federico II, Naples, Italy; ^4^SiPGI Postgraduate School in Gestalt Integrated Psychotherapy, Torre Annunziata, Italy

**Keywords:** machine learning, predictive modeling, explanatory modeling, item selection, neural networks, psychopathological assessment

## Abstract

This paper presents a procedure that aims to combine explanatory and predictive modeling for the construction of new psychometric questionnaires based on psychological and neuroscientific theoretical grounding. It presents the methodology and the results of a procedure for items selection that considers both the explanatory power of the theory and the predictive power of modern computational techniques, namely exploratory data analysis for investigating the dimensional structure and artificial neural networks (ANNs) for predicting the psychopathological diagnosis of clinical subjects. Such blending allows deriving theoretical insights on the characteristics of the items selected and their conformity with the theoretical framework of reference. At the same time, it permits the selection of those items that have the most relevance in terms of prediction by therefore considering the relationship of the items with the actual psychopathological diagnosis. Such approach helps to construct a diagnostic tool that both conforms with the theory and with the individual characteristics of the population at hand, by providing insights on the power of the scale in precisely identifying out-of-sample pathological subjects. The proposed procedure is based on a sequence of steps that allows the construction of an ANN capable of predicting the diagnosis of a group of subjects based on their item responses to a questionnaire and subsequently automatically selects the most predictive items by preserving the factorial structure of the scale. Results show that the machine learning procedure selected a set of items that drastically improved the prediction accuracy of the model (167 items reached a prediction accuracy of 88.5%, that is 25.6% of incorrectly classified), compared to the predictions obtained using all the original items (260 items with a prediction accuracy of 74.4%). At the same time, it reduced the redundancy of the items and eliminated those with less consistency.

## Introduction

Statistical modeling is traditionally separated into two different cultures. One uses an explanation-oriented approach to science, the explanatory modeling that Breiman (2001) defines as “data modeling culture.” The other uses a prediction-oriented approach, defined by Breiman as “algorithmic modeling culture.” In the former approach, data is assumed to be drawn from a given stochastic model, researchers are interested in testing the hypothesized “true” relationship between two or more variables and the mechanisms governing their intercorrelation, and the main objective is to reproduce model parameters using statistical inference and to improve the explanatory power of models. In the second approach, the data-generating process is unknown, and researchers are interested in finding an algorithm capable of recognizing different patterns hidden in data, which then gives the best prediction for the output values through the input values of new observations (Shmueli, 2010). However, in many disciplines, particularly in psychology and social sciences, statistical modeling for explanation is the predominant, if not the exclusive approach. Conversely, in domains like bioinformatics and natural language processing, algorithmic modeling is predominant (Breiman, 2001).

Beyond a confirmatory approach with the corresponding inferential assumptions (often not met in the real world), predictive modeling can help establish theoretically grounded models that have high predictive power (Sarstedt et al., 2014) and increase the efficiency and reproducibility of a researcher’s analysis (Yarkoni and Westfall, 2017). Psychology research may improve comprehensively by exploiting the potentiality of Machine Learning and Artificial Intelligence algorithms while maintaining the data modeling culture.

Psychology research needs to be grounded in a common theoretical framework of reference, which is the initial stage of the research design. The credibility of a research study is generally derived from the quality of this initial stage of the design. Consequently, psychology research should not steer toward a prediction-based orientation to the detriment of an approach that aims at testing model relationships in an explanatory sense. Even in a predictive-oriented approach, hypothesis formulation is a crucial step and it is always the investigator who chooses the statistical methods better suited for the related theoretical and empirical models. Results depend crucially on the user’s knowledge of the domain they are investigating (Pessa, 2004). In the presence of complex theories, moreover, testing a pre-determined system of hypotheses may become problematic in terms of model assumptions and interpretation. In such a case, a discovery-oriented process should be envisioned (Wold, 1985), where the investigator should be able to exploit the appropriate statistical and computational however methodologies to convert data and models into actionable insights to support such theories and for prediction purposes (Breiman, 2001; Lauro, 2019). Indeed, machine learning approaches to clinical psychology and psychiatry may focus on large multidimensional data sets to improve the decisions associated with diagnosing and treating people who have been diagnosed with mental illness using ordinary clinical methods (Dwyer et al., 2018).

In an evolved vision of the use of artificial intelligence methods in the context of psychopathology, scholars have the unprecedented opportunity to integrate complex brain, behavior and genes patterns to develop precision psychiatry. Indeed, growing evidence suggests that the classification of psychiatric patients derived from these approaches may better predict treatment outcomes than ordinary DSM/ICD-based diagnoses (Bzdok and Meyer-Lindenberg, 2018).

Another interesting use of machine learning is for demonstrating the reliability of a scale and testing for convergence validity with other variables. Instead of using traditional techniques, predictive models can achieve the same results but in a much more efficient way, computing the out-of-sample prediction accuracy of the scale with respect to one or several other measures (Du et al., 2014; Yarkoni and Westfall, 2017).

Indeed, predictive modeling can be used instrumentally to complement explanatory modeling in order to further scientific knowledge (Breiman, 2001; Shmueli, 2010; Yarkoni and Westfall, 2017; Azzolina et al., 2019). The use of the two approaches should be complementary rather than competitive. A proper combination of the two approaches may lead to the use of a wide variety of statistical and computational tools, by exploiting the strengths of both approaches through a single method in order to have stronger grounds for theory testing, knowledge discovery, prediction and decision-making, for example, for the assessment and diagnosis of psychopathology.

In line with these considerations, we think that a methodology that highlights the features of predictive modeling in terms of model building and assessment may be welcomed in psychology research and other social science disciplines, which can only benefit from these methodological developments.

The present work focuses on the psychopathological and behavioral dimensions that play the role of main nosographic organizers of psychiatric diagnosis, to improve the precision with which the classification of patients in specific diagnostic categories is carried out.

The study intends to present a new methodology for approaching prediction in a psychopathological diagnosis context applied to the construction of a novel diagnostic scale, by preserving the psychometric properties of the models as they are traditionally approached from an explicative point of view.

The current psychopathological diagnosis relies on syndromic models that we have inherited from authors such as Kraepelin and Bleuler, who operated in a pre-neuroscientific era. It follows that many psychiatric disorders are classified through obsolete concepts that do not consider the knowledge we currently have of the brain and the basic emotional systems that comprise its deepest part (Lane and Sher, 2015; Montag et al., 2017). Especially in humans, it has become increasingly evident that the phylogenetically more recent cortical structures, to which the awareness of experience links, have improved the adaptation of fundamental emotional processes to social contexts, but have not replaced the weight of emotions in the organization of social life (Panksepp et al., 2017). This evidence can have a significant impact on the psychopathological investigation that can now focuses on emotionality and affective regulation systems (Stanghellini, 2019). Indeed, the present work introduces concepts derived from affective neuroscience into psychopathological diagnostics, which up to now have been largely underestimated for the study of psychic disorders and can improve the naturalistic value and stability of psychiatric nosography.

In particular, in this paper, we propose a procedure for the selection and analysis of the items to be included in a novel scale for the evaluation of psychopathological traits based on affective neurosciences and phenomenology, which combines explanatory psychometric measurements, such as factorial coherence and construct validity, with measurements of the predictivity of the instrument carried out through machine-learning methods.

The proposed procedure identifies a well-fitting, in terms of validity and reliability of the factor structure, and a predictive yet parsimonious model among competitive ones. Indeed, parsimonious and well-fitting models exhibit higher predictive abilities and are more likely to be scientifically replicable and explainable (Sharma et al., 2019).

The main objective is to maximize the predictive ability of the model while maintaining the psychometric properties and factorial structure of the scales. A machine learning procedure is applied to identify the best predictor items for the presence of pathological variants of the personality to find the set of items that maximize the predictive ability of the model. The factorial structure is then evaluated through principal component analysis (PCA).

The model evaluation will consider the performance of the model in terms of both explanatory power and predictive accuracy. Measurements of explanatory power are typically in-sample metrics and refer to how well the proposed model (in this case, the model of the factor structure) accounts for the covariances between items. For predictive power, out-of-sample metrics are used, which are computed through a cross-validation procedure.

### Theory Reference

#### The Relationship Between Emotions and Mental Disorders

The self-report diagnostic test described in this paper is rooted in both phenomenological and neuroscientific views of emotions. In this integrated perspective, emotions present three inseparable functions: the production of socially adequate behavior; the regulation of internal homeostasis; the production of a conscious mental state characterized by adaptive values (e.g., good or bad, unpleasant or pleasant) that are salient for the subject (Maldonato et al., 2018; Sperandeo et al., 2018b).

In these functions, emotions are the basis of rational processes. As shown by numerous authors, subjects with lesions of basic emotional systems show profound impairment in their decision-making activity and are substantially incapable of responding rationally to life events (Stanghellini et al., 2016; LeDoux and Hofmann, 2018). Below we will describe the two perspectives of reading that clarify the emergence of psychopathology from affective processes in a complementary and integrable way.

For current affective neuroscience, human minds express several phylogenetically ancient emotional processes. Basic emotional tendencies have great significance for psychopathology and we consider it extremely important for the study of psychic disorders. These systems are present in all mammals but, of course, the vast cognitive capacities of humans add unique dimensions to emotional consciousness. The interweaving of cognitive and affective capacities, and in particular the aspects of memory, can make human beings particularly sensitive to psychiatric disorders. Through cognitive processes of emotional amplification, humans can sustain emotional arousal for a long time after the precipitating causes have passed. In this way, our cognitive functions can become critical agents in the creation of emotional problems. Intense emotional excitement sustained and unregulated by ruminative tendencies can interfere with our thinking patterns, even intensify, and energize our cognitive concerns by producing a deleterious vicious circle. Thanks to our remarkable cognitive abilities, we create complex mental lives, with intrapsychic tensions typical of our species. Our vast ability to look far into our memory and imagine terrible future problems pushes us to sustain the emotional excitement generated internally and to encounter psychic disorders much more than other mammals. Prolonged emotional excitement can also lead to prolonged turbulence in our bodies, producing various psychosomatic disorders and disorders in our daily quality of life (Clynes and Panksepp, 2013).

From the phenomenological perspective, emotions precisely determine the motivation for movement. They are functional states of our organism that motivate actions; they provide orientation in life by making sure that attention moves in a particular direction and attributes specific meanings and values to the world. Recognizing this aspect of emotions allows us to elevate them from mere biological reactions or mental phenomena to fundamental expressions of the “lived body,” representing the moment in which the psychobiological dimensions of experience are articulated (Messas et al., 2018; Sperandeo et al., 2019).

Emotions allow us to see reality from a specific perspective. The analysis of the mental states of an angry person and a frightened person allows us to understand the differences in their respective life perspectives. Therefore, the subject’s way of experiencing the world reflects his or her state of mind, so it follows that emotions are the primary way to understand a person and his or her psychopathology. Finally, emotions play a fundamental role in the development of sociality, inter-subjectivity and empathy. When a child perceives his mother’s happy face, he or she automatically reproduces her facial expression; through this reflection, he feels his mother’s happiness. It is an inter-corporeity produced by a perceptual-motor process, which is the very essence of the emotional phenomenon. In the absence of emotions, the world appears unreal and distant, devoid of interest and meaning. The objects that belong to the world appear to be a collection of meaningless things of which one can have a non-practical theoretical knowledge. Emotions are the motivation for performing actions, and without them, there is no motivation to move and thus no action. The absence of emotions implies the loss of vital contact with reality, everything in the world appears equivalent and devoid of salience so that neither movement, nor choice, nor meaning is possible (Stanghellini, 2019).

In our opinion, emotions – understood in their entirety as effective experiences, adaptive behaviors, and autonomous and self-regulating processes – are the basis for the emergence of psychopathological phenomena (Solms and Panksepp, 2012). The main clinical manifestations currently classified by the adult psychiatric nosography are personality disorders, pathologies resulting from mental trauma and stressful events, mood disorders, somatic symptom disorders and anxiety disorders. Negative emotions such as fear, suffering, anger are present in all of these disorders, but currently, an adequate nomenclature to describe these relationships has not agreed. Studying psychopathology from the perspective of the emotional events of a subject is therefore difficult because it cannot follow paths traced and shared in the scientific community. It is precisely for this reason that the development of an innovative vision appears to be indispensable.

#### The Panksepp Model of Emotions

In this paper, we present the development of a self-report diagnostic tool for the exploration of the psychopathological manifestations that emerge from the emotional affective processes organized in the medial part of the brain. For this purpose, we have used the model of basic emotional systems as described by Panksepp and Biven (2012). According to this approach, in mammals’ brains, there are at least seven emotional neuronal circuits (fear, rage, sexual impulses, care, anxiety of separation and social bond, playfulness, and a general system of lust and seeking) from which behaviors, autonomic processes and conscious affective states emerge which are essential for one’s interpersonal relationships.

When these systems are activated, individuals experience intense feelings, recall memories, implement behaviors of adaptation to the environment, and activate hormonal processes and vegetative regulation. The basic emotional systems at the beginning of childhood psychological development are weakly linked to the objects of the world. The basic affective tools that evolution has provided emerge in the development of the brain without an initial intrinsic connection to the events of the world. It is through life experiences, both individual and cultural that these connections are forged. Even if these emotionally evaluated systems are clustered into constellations of positive and negative affections, it seems unlikely that only two primary types of affective feelings are the raw materials from which all other affections within the brains of mammals are created. Indeed, affection is not interpreted as an independent sensory function of the brain but is based on tendencies toward action.

Considerable evidence arising from animal brain research suggests that at least seven basic emotional systems are concentrated in the subcortical regions of the brain and are located essentially in the same regions of the brain in all mammals.

A brief description of each basic emotional systems is presented below.

The SEEKING system must be conceptualized as a primary action system that helps to realize emotional drives, to seek nourishment and to realize expectations. This system operates in both positive and negative emotional situations (e.g., security seeking) and helps to maintain the fluency of the behavior as well as supporting learning and other cognitive activities (Ikemoto and Panksepp, 1999).

The FEAR system associates anxiety and the tendency to escape from the many dangers present in our world. The RAGE system supports the defense and the achievement of objectives. The LUST system supports libidinal appetites. The CARE system supports the protection and care of offspring. The GRIEF (Panic) system aims at preventing the loss of protective figures. The PLAY system aims at developing sociality (Panksepp, 2014).

## Materials and Methods

### Study Population

As part of the ordinary psycho-diagnostic evaluation procedure, 604 adult patients have been enrolled in the clinical centers of SiPGI, a specialization school in psychotherapy. The questionnaire described below was administered to subjects who agreed to participate in the study.

Personality disorders were found in 196 (32.5%) patients out of the 604. Subjects in the depressive, manic or acute psychotic phase and subjects with cognitive deficits and head injuries with detectable parenchymal lesions were excluded. The diagnosis was made using the Italian version of the personality diagnostic interviews associated with DSM-5: The Structured Clinical Interview for DSM-5 Personality Disorders (SCID-5-PD). It is one of the most used tools for the diagnosis of personality disorders in clinical and research areas and has demonstrated excellent reproducibility and clinical validity (Somma et al., 2017). The subjects that did not meet the diagnostic criteria for any nosographic category were classified as healthy, and all others were classified as unhealthy.

Characteristic of patients, as shown in Table 1, are the following: 273 males (45.2%) and 331 females (54.8%); average age of 33.96 ± 11.34, 342 (56.5%) were unmarried, 223 (36.9%) married, 32 (5.3%) divorced and 8 (1.3%) widow; 161 (26.7%) patients were graduated, 336 (55.6%) had high/secondary school, 100 (16.6%) middle school and 7 (1.2%) elementary school; 393 (65.1%) were employed, 197 (32.6%) unemployed, and 14 (2.3%) retired. No statistically significant differences between the two groups (healthy vs. unhealthy) were found for all the variables, except for marital status (*p* = 0.012).

**TABLE 1:** Characteristics of patients.

Characteristic	Total *n* = 604	Healthy *n* = 408	Unhealthy *n* = 196	*p*-Value
Sex				0.551
Male	273 (45.2%)	181 (44.4%)	92 (46.9%)	
Female	331 (54.8%)	227 (55.6%)	104 (53.1%)	
Age	33.96 ± 11.3	34.52 ± 11.4	32.78 ± 10.9	0.076
**Marital status**				
Unmarried	341 (56.5%)	129 (65.8%)	212 (52%)	0.012
Married	223 (36.9%)	58 (29.6%)	165 (40.4%)	
Divorced	32 (5.3%)	8 (4.1%)	24 (5.9%)	
Widow	8 (1.3%)	1 (0.5%)	7 (1.7%)	
**Education**				
Graduated	161 (26.7%)	50 (25.5%)	111 (27.2%)	0.962
High/secondary school	336 (55.6%)	112 (57.1%)	224 (54.9%)	
Middle school	100 (16.6%)	32 (16.3%)	68 (16.7%)	
Elementary school	7 (1.2%)	2 (1%)	5 (1.2%)	
**Occupational position**				
Employed	393 (65.1%)	124 (63.3%)	269 (65.9%)	0.789
Unemployed	197 (32.6%)	129 (31.6%)	129 (31.6%)	
Retired	14 (2.3%)	10 (2.5%)	10 (2.5%)	

*Data are reported as number of patients (%) or mean (± standard deviation), as appropriate. p-Values are based on Student’s t-test, χ^2^ test or Fisher’s exact test, as appropriate. Note that for some characteristics frequencies over categories do not sum to the total number of patients, because there were some missing values.*

### Measures

For the structuring of the questionnaire, a group of six experts in psycho-diagnostics, under the supervision of two of the authors of this work, produced a list of 260 items that – according to them – describe the dimensions of the seven basic emotional systems within the main psychic pathologies and personalities currently framed in the classification systems.

The questions are formulated to obtain dichotomous answers (yes/no), avoiding the frequency and intensity of the phenomenon under investigation within the same descriptions, limited exclusively to the detection of its presence or absence.

The items are organized into three distinct areas:

• 157 items are related to the “emotional characteristics” present in the personality disorder area. Many of these questions are presented in order to detect the non-pathological psychic phenomenon. In line with Panksepp model of basic motivational systems, most questions investigate emotional experiences and behaviors. Other questions investigate physical sensations while a small group of questions looks for the subject’s opinions to detect the impact of cortical functions on emotional systems.
• 24 questions explore the presence of “dissociative phenomena” commonly present in the area of post-traumatic pathologies. In this group of questions, only the presence of dissociative phenomena in the three dimensions of depersonalization-derealization, dissociated mental states and dissociative amnesia is sought.
• 79 questions explore the main “psychopathological traits.” These questions also explicitly refer to the presence or absence of a pathological phenomenon.

The division into three areas (emotional characteristics, dissociative phenomena, psychopathological traits) of the items arises from the theoretical assumption that the processes of sensitization or desensitization of the seven basic emotional systems produce a type of symptomatology (described in the group of items belonging to the emotional characteristics) that is different from that determined by the cognitive reworking of the emotional states (described in the group of items belonging to the psychopathological traits). Both symptoms are distinguishable from the dissociative one in which the traits of emotions produced by the system of anger and fear spread and invade the structures of awareness (Trull et al., 2015; Sperandeo et al., 2018a).

### Statistical Analysis and Multi-Step Machine Learning Procedure

Preliminary analyses concerned the handling of missing data was performed. Missing data were assumed to be missing completely at random (MCAR). The multiple imputation method for incomplete multivariate data was performed for the imputation process, using the predictive mean matching method built in the R package “mice” (Van Buuren and Groothuis-Oudshoorn, 2011).

As for the explanatory side of the work, to evaluate the factorial structure of the scales and assess its psychometric properties a PCA and orthogonal Varimax rotation was performed.

**FIGURE 1:**
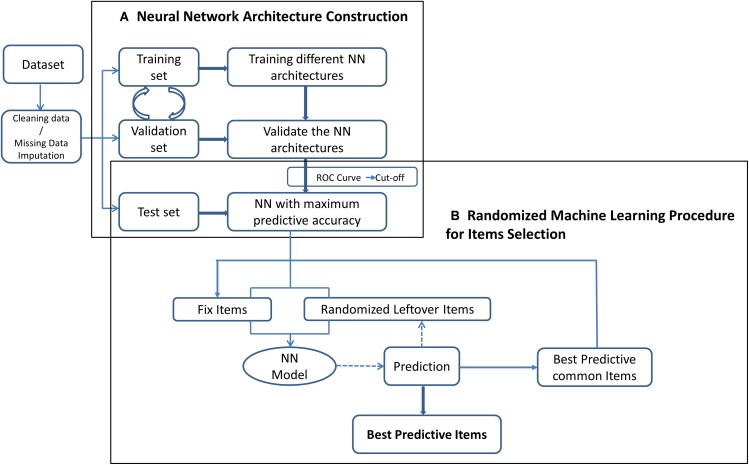
Flow chart of proposed predictive-oriented machine learning procedure.

For the predictive side, which relies on machine learning techniques, artificial neural networks (ANN) are applied as a classifier to maximize the predictive power of the model. To this end, multi-layer ANNs were trained with *resilient backpropagation algorithm* (Riedmiller, 1994) to classify subjects as healthy or unhealthy, considering all items of the scale (see Figure 1).

Resilient backpropagation (RPROP) is a fast and effective learning algorithm that uses the direction of the error gradient (i.e., the sign of the change) for calculating the weight change, rather than the actual magnitude of the partial derivative, as in the traditional backpropagation.

Resilient backpropagation calculates an individual delta Δ_*ij*_, for each connection, which determines the size of the weight update. The calculation of delta at any given time of the learning process follows the rule:

$$\Delta_{i\,j}^{t}\,\left\{ \begin{array}{cc} \eta^{+}\times \Delta_{i\,j}^{t-1},i\,f\,\frac{\partial E^{t-1}}{\partial w_{i\,j}}\times \frac{\partial E^{t}}{\partial w_{i\,j}}>0\; & \\ \eta^{-}\times \Delta_{i\,j}^{t-1},i\,f\,\frac{\partial E^{t-1}}{\partial w_{i\,j}}\times \frac{\partial E^{t}}{\partial w_{i\,j}}<0\; & \\ \Delta_{i\,j}^{t},\text{otherwise}\; & \end{array}\right.$$

where 0 < η^−^ < 1 and η>+1.

Synaptic weights ($w_{i\,j}^{t}$) are updated according the usual formula:

$$w_{i\,j}^{t}=w_{i\,j}^{t-1}+\Delta \,w_{i\,j}^{t}$$

The output neuron activation *o*_*j*_ of the ANN is calculated based on the neuron net-input *x*_*j*_, according to the following functions:

$$x_{j}=i_{i}\,w_{i\,j}-b_{j}$$

$$o_{j}=\frac{1}{1+e^{-x_{j}}}$$

where *i*_*i*_ is the i*-th* input, *b*_*j*_ is the bias of the j*-th* post-synaptic neuron and *w*_*ij*_ is the weights matrix connecting presynaptic to post-synaptic neurons.

For the actual ANN training computation we used the “neuralnet” R package (Günther and Fritsch, 2010).

The construction and the subsequent exploitation of the ANN predictive power for item selection purposes was carried out in two stages.

In a first stage, a series of fully connected ANNs with 260 input nodes (i.e., one for each item of the scale), one single output node (encoding healthy or unhealthy predictions) and a variable number of hidden units, ranging from 0 to 50, were tested. The parameters were fixed for all architectures: learning rate factors η*^–^* and η*^+^* were set at 0.5 and 1.2, respectively; synaptic weights were randomly initialized from a normal distribution in the rage [−4, 4]; the stopping criteria for the error function was 0.0005; and the maximum number of iterations was fixed in 5000 epochs. At this stage, a cross-validation procedure was used to select the best neural network architecture, i.e., the more effective number of hidden nodes, in terms of prediction accuracy. A Monte Carlo Cross-validation procedure has been chosen to avoid over-fitting in the following way: at first, from the entire set of the available data, a *test set* was extracted. In the test set we maintained the same number of patients in the two groups (healthy and unhealthy). Thus, we randomly selected the 20% of patients among unhealthy ones. Then, we selected the same number of patients among the healthy ones. Consequently, the test set was composed of about 13% of all the patients.

Subsequently, at each step of the training procedure, the remaining data were halved into two different sets: the *training set* (80% of remaining patients), which is used to find a set of good weights and bias values by comparing the desired output with the one produced by the ANN – thus for calculating the actual error – and the *validation set* (20% of remaining patients), which is used to evaluate at runtime the progress of the learning process. The *test set* is eventually used to assess the quality of the resulting ANN in terms of out-of-sample prediction accuracy at the end of the training.

Receiver operating characteristic (ROC) analysis was applied to find the optimal output node threshold, i.e., the one that gives the best diagnostic accuracy for the model (Woods and Bowyer, 1997). The (0, 1) criterion was used to select the optimal threshold, giving maximum sensitivity and specificity. This procedure assures a better prediction accuracy among groups of subjects, even if the groups are not balanced. Model performance was measured on the test data using the area under the curve (AUC) and classification error rate.

At a second stage, a knowledge-based randomized machine learning procedure was applied to identify the best predictor items for mental disorders, i.e., the set of items that maximize the predictive ability of the model. This procedure started by defining a set of items that are theoretically relevant and are never dropped from the neural network’s inputs (this is the knowledge-based part of the procedure). Then, predictions were obtained adding new items randomly sampled from the set of the remaining items. The items in common across all the “best” solutions in terms of prediction accuracy, were then considered as fixed for the following step, together with the theoretically relevant items. Then, items were again randomly sampled from the set of the remaining items until the algorithm figured out which set of items achieves the best prediction accuracy. Finally, the factorial structure of the select items was evaluated through principal PCA. The entire procedure is depicted in Figure 1.

The final model evaluation considers the performance of the model in terms of both explanatory power and predictive accuracy.

All computations and statistical analyses were performed using the R software environment for statistical computing.

## Results

### Principal Component Analysis on All Items

For all items of the scale, only the 0.1% of the data were missing and were assumed to be MCAR.

Principal component analysis was performed separately for each of the three areas, selecting seven components for each area, according to theory, because the purpose of this analysis was not to extract components, but rather to examine the coherence of the scale and the extent to which the results of the two analysis (respectively, the one with all the items and the one with only the selected items) differ.

As will be evident below, the explained variability of components appears relatively low for each area. However, it should be noted that PCA was applied to binary variables. Even though PCA on binary data provides a plausible low-dimensional representation (Gower, 1966; Jolliffe, 2002), the obtained principal components, like the components computed using multiple correspondence analysis (MCA) of categorical data, are just fractional coordinates in a smooth Euclidean space mapping, and scale indeterminacy arises. Scale change leads to the so-called low percentage of inertia problem since eigenvalues tend to zero and the variance explained by the components would be severely underestimated. Therefore, the percentage of the explained variance gives a pessimistic view of the proportion to which the extracted components account for the variation of the data and simple scale adjustment of the solution can give a more precise estimate (Benzécri, 1979; Lebart et al., 1995; Greenacre and Blasius, 2006). For these reasons, explained variance components may still be very informative, as in the case of this study, which allows us to interpret the PCA results correctly.

As shown in Table 2, for the area of “emotional characteristics presents” (136 items) the seven components cumulatively explain 25% of the variance. The first component better explains 44 items in which the “yes” answers describe a condition of hypersensitization of the system of grief. The second component represents 18 items, in which the “yes” answers describe the good functioning of the care system. The third component explains the12 items in which the “yes” answers describe a hypersensitization of the system of fear. The fourth component consists of 18 items in which the “yes” answers describe the correct functioning of the search system. The fifth component explains the 18 items in which the “yes” answers describe the good functioning of the game system. The sixth component is composed of 11 items in which the “yes” answers describe a hypersensitization of the system of anger. The seventh component is composed of 15 items in which the “yes” answers describe a hypersensitization of the system of lust.

**TABLE 2:** PCA – area of emotional characteristics.

Component n. items	Eigenvalue	% explained	Cumulative % explained variance
(1) PANIC 44 items	10.67	6.76	6.755
(2) CARE 18 items	5.31	3.36	10.114
(3) FEAR 12 items	5.31	3.36	13.472
(4) SEEK 18 items	5.27	3.34	16.809
(5) PLAY 18 items	5.27	3.34	20.144
(6) RAGE 11 items	4.75	3.00	23.15
(7) LUST 15 items	3.75	2.37	25.52

Table 3 shows the seven components selected from the items related to the area of psychopathological traits (75 items). The first component better explains 17 items in which the “yes” answers describe pathological traits determined by the hypersensitivity of the grief system. The second component is composed of 16 items in which the “yes” answers describe pathological traits determined by the hypoactivity of the Seeking system. The third component is composed of 11 items in which the “yes” answers describe pathological traits determined by the hypoactivity of the care system. The fourth component is composed of 10 items in which the “yes” answers describe pathological traits determined by the hypersensitivity of the fear system. The fifth component is composed of 12 items in which the “yes” answers describe pathological traits determined by the hyperactivity of the system of anger. The sixth and seventh components are composed of 7 and 2 items, respectively, in which the “yes” answers describe pathological traits determined by the hypoactivity of the game system and pleasure.

**TABLE 3:** PCA – area of psychopathological traits.

Component n. items	Eigenvalue	% explained variance	Cumulative % explained variance
(1) PANIC 17 items	6.408	8.215	8.215
(2) SEEK 16 items	5.454	6.992	15.208
(3) CARE 11 items	5.08	6.512	21.72
(4) FEAR 10 items	4.396	5.635	27.355
(5) RAGE 12 items	4.199	5.384	32.739
(6) PLAY 7 items	3.89	4.988	37.727
(7) LUST 2 items	1.931	2.476	40.203

Table 4 shows the two components that emerged from the area of dissociative phenomena consisting of a total of 22 items. The component of depersonalization-derealization and the composition of dissociative amnesia are composed respectively of 12 and 10 items in which the “yes” answers describe the two typical ways of altering the cognitive functions produced by the uncoordinated hyperactivity of the basic emotional systems.

**TABLE 4:** PCA – area of dissociative phenomena.

Component n. items	Eigenvalue	% explained variance	Cumulative % explained variance
(1) Depersonalization 12 items	7.83	32.6	32.6
(2) Amnesia 10 items	4.47	18.61	51.21

The group called “emotional characteristics” composed of 136 items has 15 with negative loadings, 55 with very low loadings (less than 0.4) and 38 with low loadings (less than 0.5). The component called “Seek” has 14 items 5 of them are negative. Moreover, in the component called “Panic,” there are 10 items that show significantly high values even in other components. This component, composed of 44 items, has only 8 items with high loadings (greater than 0.5).

Two out of the 75 items in the group called “psychopathological traits” are negative, 40 have very low loadings, and 26 low loadings. Twelve out of the 23 items in the group called “dissociative phenomena” have very low loadings and 4 low loadings.

### Neural Network Architecture Construction

**FIGURE 2:**
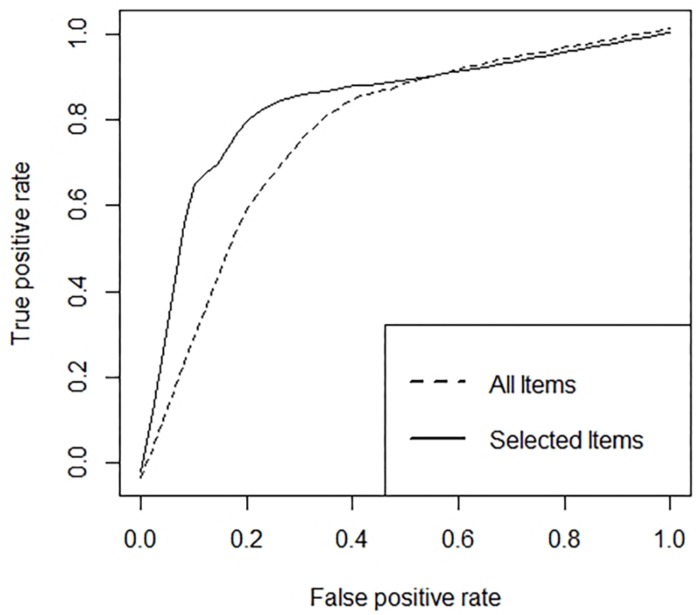
Roc curves obtained using all the items and the items selected by the proposed procedure.

As described above, a multiple-layer ANN was trained with backpropagation to classify subjects with and without the presence of pathological variants of the personality, considering all the items as inputs. The first stage of the procedure, as described in Section “Materials and Methods,” selected as the best predictive model, an ANN with 25 nodes in the hidden layer. The best result was reached in 546 epochs of training. The limit of 5000 epochs was never reached for all the architectures trained. Then, ROC analysis was applied to find the optimal threshold. The resulted threshold was 0.088. With this parameter fixed, on the out-of-sample *test set* the ANN achieved a classification error of 0.2564, meaning a prediction accuracy equal to 74.4% (i.e., 25.6% of incorrectly classified) and an AUC equal to 0.778. The corresponding ROC curve is shown as a dotted line in Figure 2. In particular, the classification error rate was equal to 28.2% for patients with the presence of pathological variants of the personality and 23.1% for patients without the presence of pathological variants of the personality.

### Knowledge-Based Randomized Machine Learning Procedure for Items Selection

The selected ANN architecture, together with the weights and the found optimal threshold, was then used for the item selection procedure. Predictions and classification error rates were computed using only the *test set*.

Twenty-one items were chosen as theoretically relevant and therefore, always considered as fixed inputs of the ANN. These items are descriptive of (a) mood disorders both in the depressive and manic sense, (b) alterations of the content of thought and (c) dis-perceptive phenomena. They were chosen for their fundamental link with psychopathology.

A multi-step procedure was needed to find the optimal solution, i.e., the set of items that achieves the best prediction accuracy. At the first step, 5 million combinations of items were randomly sampled from the set of the remaining items. Then, the items that appear in the solution with the lowest classification error rate were selected and added up to theoretically relevant items (thus, both sets were considered as fixed inputs for the subsequent steps). At the second step, another 5 million combinations of items were randomly sampled from the set of remaining items and the common items across all the “best” solutions were again selected and considered as fixed items for the subsequent steps. This procedure was repeated until the classification error rate of the “best” solution did not improve.

The entire selection procedure took about 10 h to complete on a parallel implementation of R running over 2 processors on a Windows 10 Pro 64-bit platform equipped with a i5-7200u intel processor and 8 GB of RAM.

**FIGURE 3:**
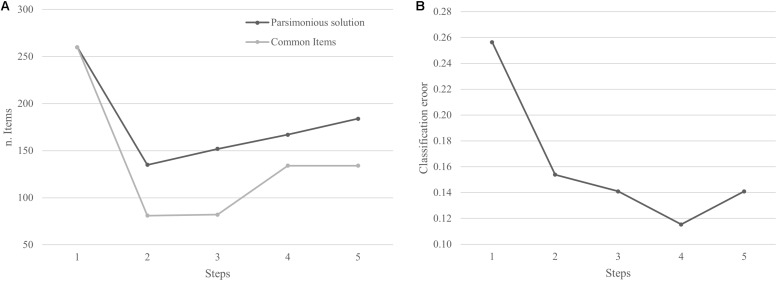
**(A)** Number of Items for the best solution and number of common items at each step. **(B)** Classification error at each step.

Figure 3 represents the number of items for the best-parsimonious solution and (a) the number of common items obtained at each step and (b) the corresponding classification error. It clearly shows that the best solution is reached in four steps. Solutions after the 4^th^ step are all worse (data not shown).

At step 4, the best prediction accuracy was achieved by a combination of 167 items, the 21 theoretically relevant ones and 146 selected by the randomized machine learning procedure. Among the selected items, 98 items relate to emotional characteristics, 15 relate to dissociative phenomena and 33 relate to psychopathological traits.

The prediction accuracy on the test set was equal to 88.5% (i.e., 11.5% incorrectly classified) and an AUC equal to 0.849. The corresponding ROC curve is shown as a solid line in Figure 2. In particular, the classification error rate was equal to 15.4% for patients with the presence of pathological variants of the personality and 7.7% for patients without the presence of pathological variants of the personality.

### Principal Component Analysis on the Selected Items

Table 5 shows the results of the PCA performed on the group of items (78 items) in the area of emotional characteristics selected by the neural network. The seven components from the PCA (globally explaining 28.7% of the variance) appear to be perfectly consistent with the reference theory discussed in the previous sections. The items of the specific components describe behaviors and affective mental contents provided in the Panksepp model. The first component explains 23 Items in which the “yes” answers describe a condition of hypersensitization of the system of grief. People described by these items tend to be blocked by a continuous state of anguish that annihilates them and leads them into a state of depression. The second component consists of 12 items in which the “yes” answers describe the good functioning of the care system. The people described in these items know how to take care of others and the system to which they belong. The third component is composed of 9 items in which the “yes” answers describe a hypersensitization of the seeking system. The people described by this component are optimistic, open to seeking and focused on achieving their goals. The fourth component consists of 14 items in which the “yes” answers describe how well the play system works. The people described by this component can socialize and enjoy the experiences of life. The fifth component is composed of 8 items in which the “yes” answers describe a hypersensitization of the system of lust. The people described by these items live in the continuous fantasy of satisfying their libidinal urges. The sixth component is composed of 5 items in which the “yes” answers describe a hypersensitization of the system of anger. The people described by these items are intolerant and aggressive. The seventh component is composed of 7 items in which the “yes” answers describe a hypersensitization of the system of fear. The people described by this component exert control over their world because they have associated numerous dangers with the activation of this emotional system.

**TABLE 5:** PCA loadings – Area of emotional characteristics in the pool of selected items (28.67% of explained variance – Kaiser-Meyer-Olkin = 0.839).

**PANIC (Items = 23, Eigenvalue = 11.05,% explained variance = 8.57)**	
è insicuro d’avanti ai problemi?	0.62
rinuncia facilmente alle cose perché si preoccupa dei rischi?	0.588
quando è stanco ha bisogno (o chiede aiuto) agli altri?	0.551
si sente inferiore agli altri?	0.528
deve impegnarsi molto per avere fiducia in se stesso?	0.519
attende che gli altri le risolvano i problemi	0.507
ha paura che le sue cose vadano male?	0.5
fa fatica a guarire da un malanno?	0.5
pensa di avere problemi al cervello?	0.492
la sua vita è priva di senso?	0.49
è pessimista?	0.479
ha bisogno di riposare durante la giornata?	0.444
rinuncia facilmente difronte a compiti impegnativi?	0.441
è preoccupato difronte a situazioni nuove?	0.411
fa fatica a comprendere le persone?	0.41
ha cattive abitudini che vorrebbe cambiare?	0.409
la preoccupano gli imprevisti?	0.409
le sue scelte sono determinate dagli altri?	0.365
ignora quale sia lo scopo della sua vita?	0.358
si entusiasma lentamente per le novità?	0.355
quando fa degli errori se la cava da solo?	0.339
si sente carico di energia per tutta la giornata?	–0.427
è molto sicuro di se?	–0.496
**CARE (Items = 12, Eigenvalue = 4.83,% explained variance = 3.74, cumulative% explained variance = 12.31)**	
è connesso spiritualmente agli altri?	0.597
ha mai avuto esperienze paranormali?	0.518
ha mai fatto intense esperienze spirituali?	0.495
sente un legame profondo con la natura?	0.493
quando è concentrato molto perde la cognizione del tempo e dello spazio?	0.471
è talmente preso dalle sue attività da perdere il contatto con la realtà?	0.429
ha idee creative quando si lascia andare all’ozio?	0.396
gli altri la definiscono distratto?	0.332
la vita dipende da una forza spirituale al di sopra di noi?	0.33
è accomodante con gli altri?	0.329
sa di avere un ‘sesto senso’?	0.321
è costante nelle cose che fa?	0.307
**SEEK (Items = 9, Eigenvalue = 4.75,% explained variance = 3.68, cumulative% explained variance = 15.99)**	
si definirebbe ottimista?	0.495
inventa storie o dice bugie solo per divertimento?	0.467
è tranquillo sul suo futuro?	0.436
evita situazioni o attività che la irritano?	0.408
le sono indifferenti i complimenti?	0.387
sa mentire bene?	0.361
ritiene importante i legami di amicizia?	0.35
è a suo agio anche con persone sconosciute?	0.331
affronta le difficoltà prendendole come sfide?	0.309
**PLAY (Items = 14, Eigenvalue = 4.66,% explained variance = 3.61, cumulative% explained variance = 19.61)**	
soffre se vede altri soffrire?	0.556
tende ad aiutare gli altri?	0.51
ama collaborare con gli altri	0.491
tende a collaborare con gli altri?	0.449
è empatico e disponibile?	0.436
è altruista anche con chi l’ha trattata male?	0.392
reagisce agli eventi coerentemente con i suoi valori?	0.389
riflette molto prima di prendere una decisione?	0.388
trova qualcosa di poetico anche nelle piccole cose?	0.387
agisce secondo le sue abitudini?	0.359
ha molte buone abitudini quotidiane?	0.354
si commuove davanti a prodotti artistici?	0.336
investe molta energia per fare le cose?	0.329
sta male se perde delle amicizie?	0.324
**LUST (Items = 8, Eigenvalue = 4.28,% explained variance = 3.32, cumulative% explained variance = 22.92)**	
desidererebbe essere più bello di chiunque altro?	0.57
le piacerebbe essere il più intelligente di tutti?	0.568
vorrebbe essere più potente di chiunque altro?	0.56
le piacerebbe essere il più forte di tutti?	0.512
le piace fare shopping?	0.422
le piacerebbe non-invecchiare mai	0.381
le piacerebbe fermare il tempo?	0.372
abbandona facilmente se non è sicuro di ottenere ciò che vuole?	0.306
**RAGE (Items = 5, Eigenvalue = 3.91,% explained variance = 3.03, cumulative% explained variance = 25.95)**	
non-tollera chi la pensa diversamente da lei?	0.516
è intollerante nei confronti di chi è diverso da lei?	0.51
si spazientisce quando gli altri non-sono d’accordo con lei?	0.485
impone agli altri il suo modo di fare le cose?	0.427
è molto fortunato/a	0.339
**FEAR (Items = 7, Eigenvalue = 3.51,% explained variance = 2.72, cumulative% explained variance = 28.67)**	
Tende a risparmiare molto?	0.436
tende a nascondere le sue emozioni?	0.427
ha difficoltà ad aprirsi con gli amici?	0.424
riflette a lungo su ciò che è giusto e ciò che è sbagliato?	0.383
riflette intensamente prima di decidere?	0.368
tende generalmente a risparmiare denaro?	0.342
Mantiene il controllo delle sue emozioni?	0.307

Table 6 shows the seven components that emerged from the area of psychopathological traits selected from the neural network, which is composed of 68 items. The first component is composed of 17 items in which the “yes” answers describe pathological traits determined by the hypersensitization of the grief system. The people described in this component can be self-destructive and hetero-destructive. The second component consists of 12 items in which the “yes” answers describe pathological traits determined by the hypofunction of the care system. The people described here are unable of taking care of their environment and the people around them, who they feel to be dangerous and intrusive.

**TABLE 6:** PCA loadings – Area of psychopathological traits in the pool of selected items (40.78% of explained variance – Kaiser-Meyer-Olkin = 0.941).

**PANIC (Items = 17, Eigenvalue = 6.47,% explained variance = 8.29)**	
le persone non le sono amiche?	0.657
si accorge che gli altri la guardano e/o parlano male di lei?	0.621
gli altri non-apprezzano il suo lavoro?	0.609
ha un senso di fastidio se gente la guarda?	0.547
ha idee che nessuno condivide?	0.541
si sente incompreso?	0.54
ha l’impressione che gli altri si approfittino di lei?	0.539
è sensibile alle critiche e alle offese	0.511
ha scarsa fiducia negli altri?	0.496
critica facilmente la gente?	0.484
si sente inferiore agli altri o inadeguato?	0.457
è imbarazzato in presenza di altre persone?	0.47
Pensa che alcune persone sono responsabili dei malesseri che prova?	0.417
si sente a disagio quando è in compagnia?	0.406
litiga spesso con le persone?	0.402
è timido verso le persone di sesso opposto?	0.39
pensa di stare scontando una pena?	0.315
**CARE (Items = 12, Eigenvalue = 5.49,% explained variance = 7.04, cumulative% explained variance = 15.33)**	
è incapace di portare a termine un compito?	0.559
si sente la mente vuota?	0.551
ogni cosa le richiede uno sforzo?	0.546
trascura cose importanti della sua vita?	0.516
si sente inutile?	0.486
ha problemi di memoria?	0.485
ha difficoltà a prendere decisioni?	0.475
ha scarsi interessi?	0.44
si sente senza speranza?	0.438
ritiene di dover sempre finire ciò che ha iniziato?	0.431
si colpevolizza facilmente?	0.426
si sente lontano dalle altre persone?	0.383
**SEEK (Items = 13, Eigenvalue = 4.92,% explained variance = 6.31, cumulative% explained variance = 21.6)**	
ha dolori muscolari?	0.667
si sente fisicamente debole?	0.661
soffre di mal di schiena?	0.612
ha gli arti appesantiti?	0.598
ha nausea o mal di stomaco?	0.561
si affatica facilmente?	0.478
ha palpitazioni o cuore in gola?	0.476
passa rapidamente da sensazioni di freddo a sensazioni di caldo?	0.468
ha un nodo alla gola?	0.464
affatica facilmente?	0.458
le capita di sentirsi venir meno?	0.432
pensa di avere una grave malattia fisica o mentale?	0.4
soffre di cefalea?	0.396
**FEAR (Items = 8, Eigenvalue = 4.85,% explained variance = 6.22, cumulative% explained variance = 27.86)**	
evita alcuni oggetti. situazioni o luoghi perché la spaventano?	0.709
ha paura di viaggiare su un mezzo di trasporto	0.671
ha paura di uscire da solo?	0.643
ha dei momenti di terrore o panico	0.582
ha paura di tutto senza un valido motivo?	0.536
ha paura?	0.525
si sente a disagio tra la folla?	0.488
è a disagio quando è solo?	0.414
**RAGE (Items = 10, Eigenvalue = 4.02,% explained variance = 5.15, cumulative% explained variance = 33)**	
sente l’impulso di distruggere le cose?	0.628
si arrabbia tanto?	0.556
sente l’impulso a colpire o a far male a qualcuno?	0.523
rompe oggetti e grida facilmente?	0.501
ha dei pensieri che non-sono suoi?	0.495
alcune persone controllano i suoi pensieri?	0.489
pensa al suicidio?	0.475
sente voci o rumori che altri non-sono in grado di sentire?	0.465
alcune persone percepiscono il suo pensiero	0.44
ha la sensazione di essere preso in trappola?	0.415
**PLAY (Items = 5, Eigenvalue = 3.17,% explained variance = 4.07, cumulative% explained variance = 37.8)**	
è insofferente e irritato?	0.594
è una persona nervosa?	0.47
si preoccupa facilmente per qualsiasi cosa?	0.434
si sente triste?	0.418
è teso o sulle spine?	0.401
**LUST (Items = 3, Eigenvalue = 2.49,% explained variance = 3.19, cumulative% explained variance = 40.27)**	
ha scarso appetito?	0.578
piange facilmente?	0.438
si sente solo anche se è in compagnia di altre persone?	0.402

The third component is composed of 13 items in which the “yes” answers describe pathological traits determined by the hypoactivity of the seeking system. The people described in these items are basically incapable of activating themselves to satisfy their desires and feel life as a strenuous physical effort.

The fourth component is composed of 8 items in which the “yes” answers describe pathological traits determined by the hypersensitivity of the fear system. The people described by these items are continuously in a state of anxiety and defense from dangers. The fifth component is composed of 10 items in which the “yes” answers describe pathological traits determined by the hyperactivity of the rage system. The sixth component is composed of 5 items in which the “yes” answers describe pathological traits determined by the hyposensitivity of the play system. The people described by these items are incapable of adequate socialization. The seventh component consists of 3 items and describes people with a hyperactivity of the pleasure system.

Table 7 shows the area of dissociative phenomena composed of a total of 15 items selected from the neural network divided into three components. Although three components have emerged, composed of 6, 5, and 4 items respectively, they describe depersonalization/disorganization and dissociative amnesia, two typical ways of altering the cognitive functions produced by the intrusion of emotionality into conscious experiences.

**TABLE 7:** PCA loadings – Area of dissociative phenomena in the pool of selected items (40.55% of explained variance – Kaiser-Meyer-Olkin = 0.931).

DEPERSONALIZATION (Items = 6, Eigenvalue = 3.7,% explained variance = 15.41)	
le capita di vedere il modo come se fosse attraverso un vetro?	0.727
le capita di sentirsi una persona diversa da come normalmente è?	0.629
le è capitato di sentire come sconosciuti i luoghi che le sono familiari?	0.617
ha mai sentito i suoi sogni come se fossero reali?	0.607
le è capitato di non-riconoscere la sua immagine allo specchio?	0.585
sente nella testa voci che commentano i suoi pensieri eı/o le	0.511
dicono cosa fare?	
A**MNESIA (Items = 5, Eigenvalue = 3.63,% explained variance = 15.11, cumulative% explained variance = 30.52)**	
le capita di non-sapere se ha fatto una cosa o se ha solo pensato di farla?	0.761
si è accorto aver fatto cose che non-ricordava di aver fatto?	0.721
le capita di possedere oggetti che non-ricorda di aver acquistato?	0.497
le è capitato di rivivere eventi già vissuti?	0.436
incontra persone che la conoscono ma che lei non-riconosce?	0.409
**IMAGINATIVE ABSORPTION (Items = 4, Eigenvalue = 2.41,% explained variance = 10.03, cumulative% explained variance = 40.55)**	
le è capitato di non-riconoscere persone che le sono familiari?	0.785
le capita di accorgersi di essersi vestito senza ricordarsi di averlo fatto?	0.563
ha dimenticato eventi importanti nella sua vita?	0.561
le capita di trovarsi in luoghi che non-ricorda di aver raggiunto?	0.361

The tables mentioned above present the items in the Italian language, as the original and only language of the diagnostic scale is Italian. For the benefit of not Italian speakers, an English translation of the selected items is provided in the Supplementary Appendix. However, it should be noted that the English version provided has never been validated nor used with English speaking subjects and it is only intended as language aid. Moreover, the items presented and translated do not sum up to 167, as previously indicated, as 6 of them did not load on any component and were discarded.

In addition to the strict consistency of the components with theoretical reference model, the items that make up the components of each of the three areas have a marked internal consistency as documented by a Cronbach α value of 0.900 for the area of emotional characteristics, of 0.889 for the area of dissociative phenomena and alfa value of 0.953 for the area of psychopathological traits.

Only 2 out of the 78 items in the group called “emotional characteristics” have negative loadings, 32 have very low loadings and 26 low loadings. No items have high values in more than one component.

One out of the 51 items in the group called “psychopathological traits” is negative, only 4 have very low loadings and 11 low loadings. Only 2 out of the 15 items in the group called “dissociative phenomena” have low loadings.

## Conclusion

In this work, we have presented a procedure that aims at combining explanatory and predictive modeling for the construction of novel psychometric questionnaires based on psychological and neuroscientific theoretical grounding, especially with regards to the aspect of the item selection, in a way that considers both the explanatory power of the theory and the predictive power of modern computational techniques. Such combination allows deriving theoretical insights on the characteristics of the items selected and their conformity with the theoretical framework of reference. At the same time, it permits the selection of items that have the most relevance in terms of prediction by therefore considering the relationship of the items with the actual psychopathological diagnosis, helping to construct a diagnostic tool that both conforms with the theory and with the individual characteristics of the population at hand, by providing insights on the power of the scale in precisely identifying out-of-sample pathological subjects.

The proposed randomized machine learning procedure selected a set of items that drastically improved the prediction accuracy of the model, compared to the predictions obtained using all the original items. At the same time, it reduced the redundancy of the items and eliminated those with less consistency.

Moreover, comparing the results obtained applying PCA on all the items and the results obtained using only the set of items selected by the ANN, clear differences emerge in the distribution and consistency of the items among the different components. The hypothesized latent structure is indeed only partially confirmed by the analysis of all items of the test. However, on the group of selected items, it clearly emerges a greater coherence in the components obtained by the PCA, better confirming the hypothesized latent structure.

The methodology exploits the relationships and the inner consistency that link the theoretical assumptions and the experience of the psychopathology, by showing that focusing on the prediction of the diagnosis and the pathology phenomena can also help to support the explanatory modeling of those phenomena.

By looking at the relationship between the items selected by the procedure and the proposed theoretical framework, by following the psychopathological model identified, it is consistent that some systems produce adaptation problems if they are hyperactive (for example the panic systems of fear and anger produce malaise only if they are active) and other systems are maladaptive if hypoactive. Such dynamic is captured by the “yes” or “no” answers within the questionnaire.

The components that emerge from the group of “emotional characteristics” describe maladaptive processes that are expressed at a non-verbal level of consciousness and do not require the intervention of cortical functions of judgment or conscious evaluation of events (Solms and Panksepp, 2012). In our opinion they can represent the emotional substrate of personality disorders.

The selected items that belong to the group “psychopathological traits” describe maladaptive phenomena that require the intervention of cognitive evaluation and belong to that group of behaviors, psychic functions, emotional states and contents of thought unanimously considered as psychiatric symptoms. In our opinion, the components that emerged in this group describe the action of the conscious mind on basic emotional states. This group of components can represent the emotional dimension of the psychopathology of mental disorders (Panksepp, 2014).

The items belonging to the group “dissociative phenomena” present three components that describe the destructuring of the self-experience and episodic memory. This psychopathological manifestation is due to traumatic events that can occur in every moment of the person’s life acutely and intensely or with less intensity for a very long time (Lanius et al., 2014).

In conclusion, we believe that the present methodology has the potential to offer an approach for the construction of new psychometric scales or the reorganization of existing ones, by focusing on the predictive power of the scale in accordance with observable phenomena, in conjunction with the traditional dimensional approach that characterizes many modern psychometric tools.

In the exemplar case presented in this work, we are aware that additional investigations are required for a compelling validation of the proposed psychometric questionnaire, to demonstrate its robustness further and support its use in real psychodiagnostic settings. At the same time, the methodology could be likewise applied to the restructuring of existing and already validated psychometric scales. This work, envisioned for the future, might further support the validity of such methodology. Moreover, we will try to combine predictive and validity metrics in a unified procedure to balance the validity and predictive performance of models, toward the definition of prediction-based validity principls and tools. Nevertheless, we believe that its application to scale constructions, as in the present case, might already demonstrate the potential of the proposed approach.

## Data Availability Statement

The datasets generated for this study are available on request to the corresponding author.

## Ethics Statement

The studies involving human participants were reviewed and approved by Ethical Committee of Psychological Research of the Department of Humanistic Studies, University of Naples Federico II. The patients/participants provided their written informed consent to participate in this study. Patients agreed to participate in the study, then signed the consent form currently required by Italian law and were informed that the data collected would be treated anonymously and would not result in changes in their diagnostic and therapeutic course of treatment.

## Author Contributions

PD: methodology design, methodology implementation, running experimentation, and writing technical sections of the manuscript and results. DM: methodology design, contribution on methodology implementation, data analysis, running experimentation, and contribution in writing technical sections of the manuscript and results. MM: design and implementation of psychodiagnostic tool, contribution in methodology design, contribution in data collection, and contribution in writing psychological sections of the manuscript. RS: design and implementation of psychodiagnostic tool, contribution in methodology design, data collection, data analysis, and writing psychological sections of the manuscript.

## Conflict of Interest

The authors declare that the research was conducted in the absence of any commercial or financial relationships that could be construed as a potential conflict of interest.
