# Supplementary material for: Toward a Machine Learning Predictive-Oriented Approach to Complement Explanatory Modeling. An Application for Evaluating Psychopathological Traits Based on Affective Neurosciences and Phenomenology
Source: Front Psychol. 2020 Mar 24;11:446. doi: 10.3389/fpsyg.2020.00446 (PMC7105860; doi:10.3389/fpsyg.2020.00446)
Supplement: Supplementary file 1 [file Data_Sheet_1.docx]

**Appendix**

**Items in English**

| 1. Are you unsure of the problems? | 1. Does it bother you if people look at you? |
| --- | --- |
| 1. Do you give up things easily because you care about the risks? | 1. Do you have ideas that nobody shares? |
| 1. When you are tired, do you need (or ask for help) from others? | 1. Do you feel misunderstood? |
| 1. Do you feel inferior to others? | 1. Do you get the impression that others take advantage of you? |
| 1. Does he have to work hard to have self-confidence? | 1. You are sensitive to criticism and insults. |
| 1. She waits for others to solve her problems | 1. Have you little faith in others? |
| 1. Are you afraid that your things will go wrong? | 1. Do you criticize people easily? |
| 1. Is it difficult to recover from ailment? | 1. do you feel inferior to others or inadequate? |
| 1. Do you think you have brain problems? | 1. Are you embarrassed in the presence of other people? |
| 1. Is your life meaningless? | 1. Do you think that some people are responsible for your illness? |
| 1. Are you pessimistic? | 1. Do you feel uncomfortable when you are in company? |
| 1. Do you need to rest during the day? | 1. Do you often quarrel with people? |
| 1. Do you easily give up in front of demanding tasks? | 1. Are you shy towards people of the opposite sex? |
| 1. Are you worried about new situations? | 1. Do you think you are serving a sentence? |
| 1. Is it hard for you to understand people? | 1. Are you incapable of completing a task? |
| 1. Do you have bad habits that you would like to change? | 1. does your mind feel empty? |
| 1. Are you worried about unexpected events? | 1. does everything require an effort? |
| 1. Are your choices determined by others? | 1. Do you neglect important things in your life? |
| 1. Do you know what the purpose of your life is? | 1. Do you feel useless? |
| 1. Do you slowly get excited about the news? | 1. Do you have memory problems? |
| 1. When do you make mistakes do it yourself? | 1. Do you have trouble making decisions? |
| 1. Do you feel energized all day long? | 1. Do you have little interest? |
| 1. Are you very sure of yourself? | 1. Do you feel hopeless? |
| 1. Are you spiritually connected to others? | 1. Do you always feel you have to finish what you started? |
| 1. Have you ever had paranormal experiences? | 1. Do you blame yourself easily? |
| 1. Have you ever had intense spiritual experiences? | 1. Do you feel far away from other people? |
| 1. Do you feel a deep connection with nature? | 1. does he have muscle pain? |
| 1. When he is very concentrated, does he lose track of time and space? | 1. Do you feel physically weak? |
| 1. Are you so busy with yours activities that you loses contact with reality? | 1. Do you suffer from back pain? |
| 1. Do you have creative ideas when you let yourself go | 1. Are your limbs heavy? |
| 1. Do others call you absent-minded? | 1. Do you have nausea or stomach pain? |
| 1. life depends on a spiritual force above us? | 1. Does he get tired easily? |
| 1. Are you accommodating with others? | 1. Do you have palpitations or a heart in your throat? |
| 1. You know you have a sixth sense? | 1. Do you quickly change from cold to hot sensations? |
| 1. Are you constant in the things you do? | 1. Do you have a knot in your throat? |
| 1. Would you call yourself an optimist? | 1. Does it tire easily? |
| 1. Do you make up stories or just lie for fun? | 1. Does it feel like it's failing? |
| 1. Are you calm about your future? | 1. Do you think you have a serious physical or mental illness? |
| 1. Do you avoid situations or activities that irritate you? | 1. Do you suffer from headaches? |
| 1. Are compliments indifferent to you? | 1. does she avoid certain objects, situations or places because they frighten her? |
| 1. Do you lie well? | 1. Are you afraid to travel on a means of transport |
| 1. Do you consider friendship ties important? | 1. are you afraid to go out alone? |
| 1. Are you comfortable with unknown people too? | 1. Have you moments of terror or panic |
| 1. Do you face difficulties by taking them as challenges? | 1. Are you afraid of everything for no good reason? |
| 1. Do you suffer if you see others suffering? | 1. Are you afraid? |
| 1. Do you tend to help others? | 1. Do you feel uncomfortable in a crowd? |
| 1. You love to collaborate with others | 1. Are you uncomfortable when you're alone? |
| 1. Do you tend to collaborate with others? | 1. do you feel the urge to destroy things? |
| 1. Are you empathetic and helpful? | 1. Do you get that angry? |
| 1. Are you selfless even with those who have treated you badly? | 1. Do you feel the urge to hit or hurt someone? |
| 1. Do you react to events consistently with its values? | 1. Do you break things and shout easily? |
| 1. Do you reflect long before making a decision? | 1. Do you have thoughts that aren't his own? |
| 1. Do you find something poetic even in small things? | 1. Do some people control his thoughts? |
| 1. Do you act according to your habits? | 1. Do you think about suicide? |
| 1. Do you have many good daily habits? | 1. Do you hear voices or noises that others can't hear? |
| 1. Are you moved by artistic products? | 1. Some people perceive your thoughts. |
| 1. Do you invest a lot of energy to do things? | 1. Do you feel like he's being trapped? |
| 1. Are you sick if you lose friends? | 1. Are you intolerant and irritated? |
| 1. Would you like to be more beautiful than anyone else? | 1. Are you a nervous person? |
| 1. Would you like to be the smartest of all? | 1. Do you easily worry about anything? |
| 1. Would you like to be more powerful than anyone else? | 1. Do you feel sad? |
| 1. Would you like to be the strongest of all? | 1. Are you tense or on edge? |
| 1. Do you like shopping? | 1. Have you a little appetite? |
| 1. Would you like never to grow old? | 1. Do you cry easily? |
| 1. Would you like to stop time? | 1. Do you feel lonely even though he's in the company of other people? |
| 1. do you gives up easily if you are not sure he gets what you want? | 1. Do you happen to see the way as if it were through a glass? |
| 1. Do you not tolerate those who think differently from you? | 1. Do you happen to feel a different person than you normally are? |
| 1. Are you intolerant of those who are different from you? | 1. Did you happen to feel familiar places like strangers? |
| 1. Do you get impatient when others don't agree with you? | 1. Have you ever felt your dreams as if they were real? |
| 1. Does it impose its way of doing things on others? | 1. Did you not recognize his image in the mirror? |
| 1. You are very lucky | 1. Do you hear voices in your head that comment on your thoughts and / or tell you what to do? |
| 1. Do you tend to save a lot? | 1. Do you not know if you did something or if you only thought of doing it? |
| 1. Do you tend to hide your emotions? | 1. Are you aware you did things you didn't remember doing? |
| 1. Do you find difficult to open up with friends? | 1. Do you happen to own items that you don't remember buying? |
| 1. Do you long reflect on what is right and what is wrong? | 1. Did you happen to relive events already experienced? |
| 1. Do you think hard before you decide? | 1. Do you meet people who know you but don't recognize you? |
| 1. Do you generally tend to save money? | 1. Did you not recognize people you are familiar with? |
| 1. Do you keep control of your emotions? | 1. Do you happen to notice that you have dressed without remembering that you did it? |
| 1. people aren't friends with you? | 1. Have you forgotten important events in your life? |
| 1. do you notice that others look at you and/or speak ill of you? | 1. Do you happen to be in places you don't remember reaching? |
| 1. Others don't appreciate your work? |  |
